# Supplementary material for: Critical assessment of missense variant effect predictors on disease-relevant variant data
Source: Hum Genet. 2025 Mar 21;144(2-3):281–93. doi: 10.1007/s00439-025-02732-2 (PMC11976771; doi:10.1007/s00439-025-02732-2)
Supplement: Supplementary file 1 — (pdf 1384 KB) [file 439_2025_2732_MOESM1_ESM.pdf]

Supplementary Figures

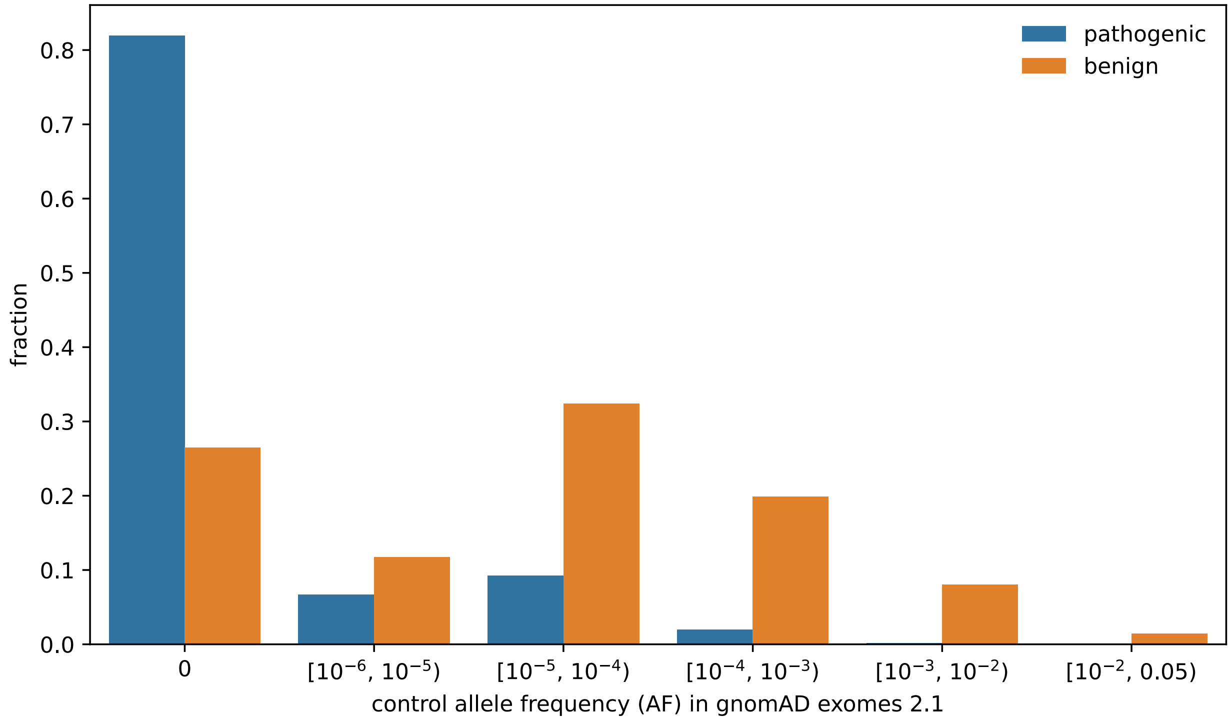

Fig. S1: **Allele frequency distributions of pathogenic and benign variants in the full evaluation dataset.** Allele frequencies were obtained for all variants from the control cohort exomes in gnomAD v2.1.1 [15].

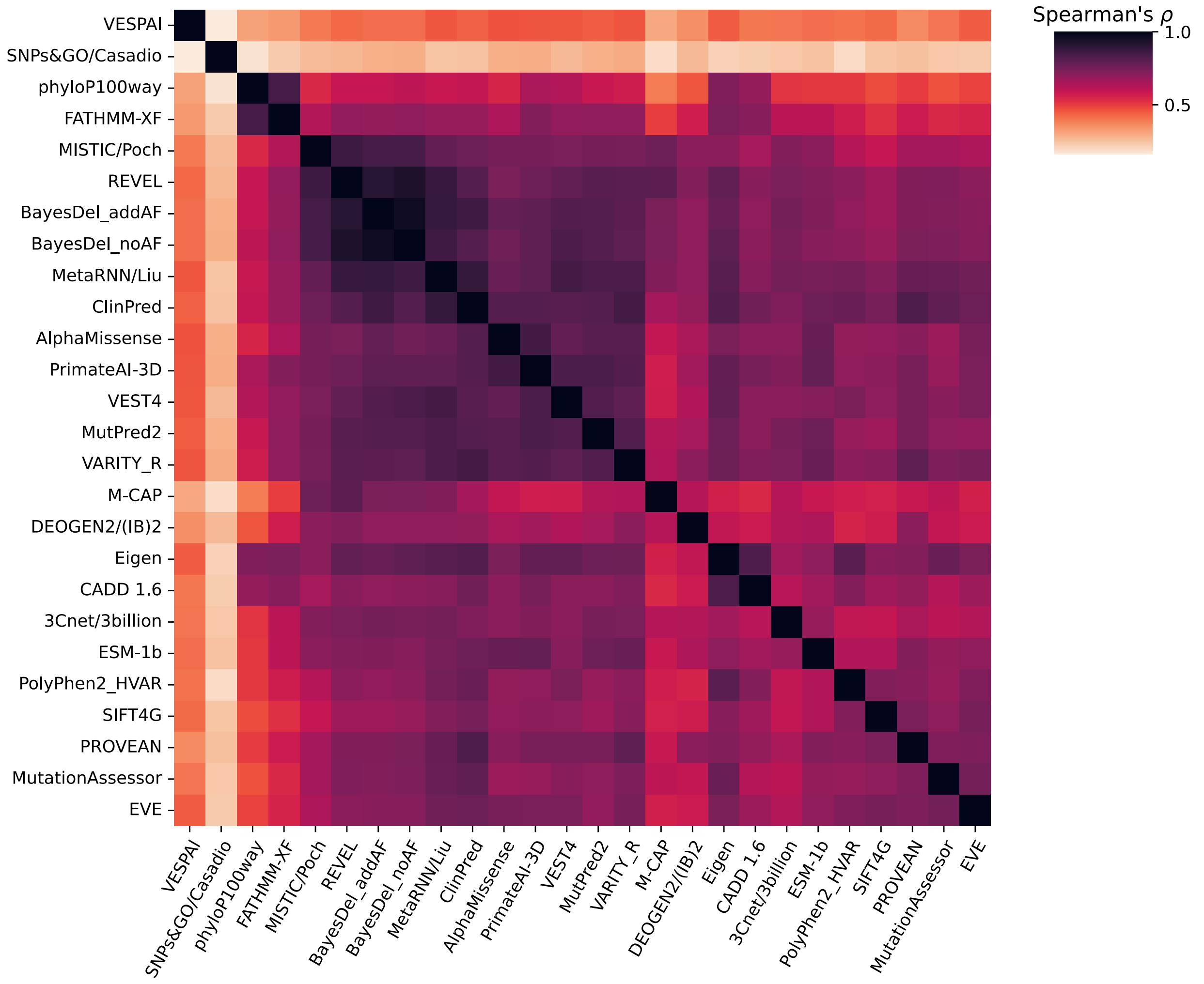

Fig. S2: **Correlations between the predictions of different tools.** The heatmap shows the Spearman rank correlation coefficients between predictions computed on variants in the full evaluation dataset.

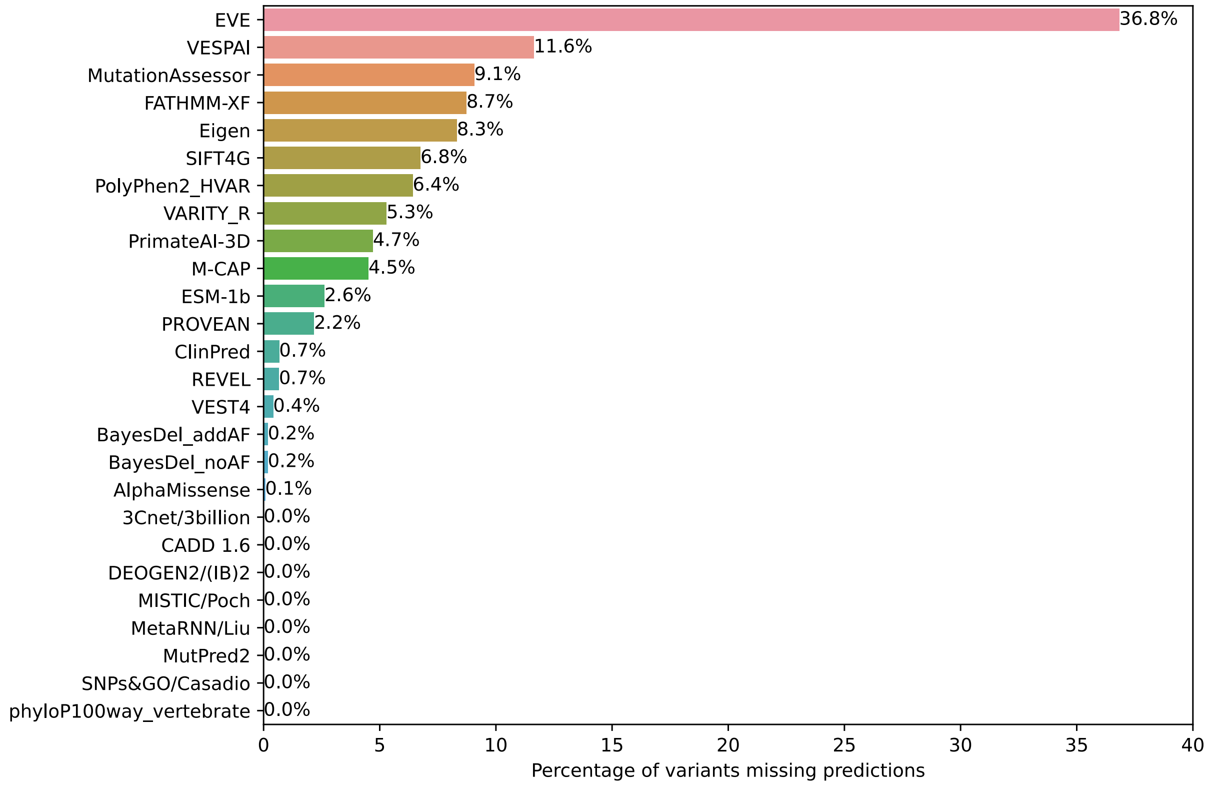

Fig. S3: **Missing predictions on evaluated variants.** The percentage of variants within the full evaluation dataset for which predictions are not provided by each tool.

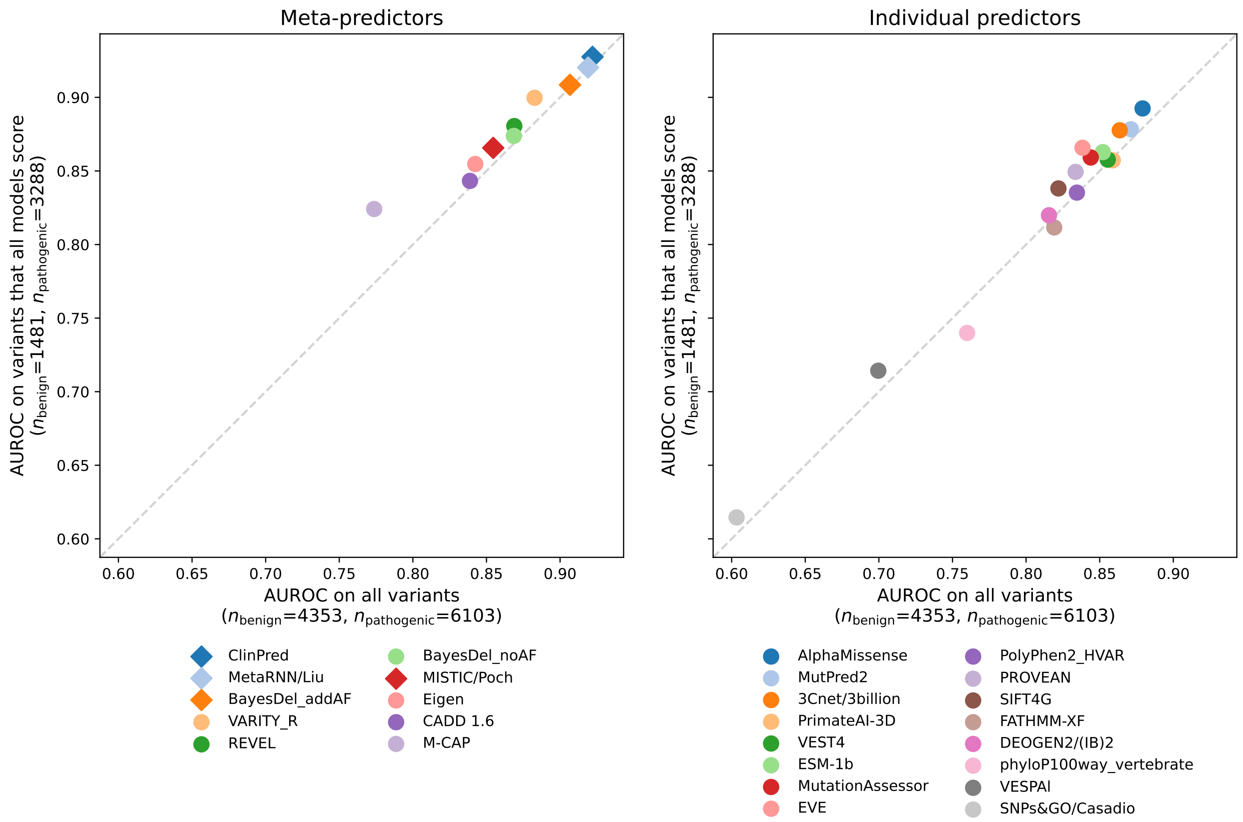

Fig. S4: **Performance on the subset of variants scored by all predictors.** We compare AUROCs on the full set of variants (Fig. 1; x-axis) to AUROCs on the subset of variants scored by all predictors (y-axis) for meta-predictors (left) and individual predictors (right). Predictors marked by diamonds use allele frequency as a feature.

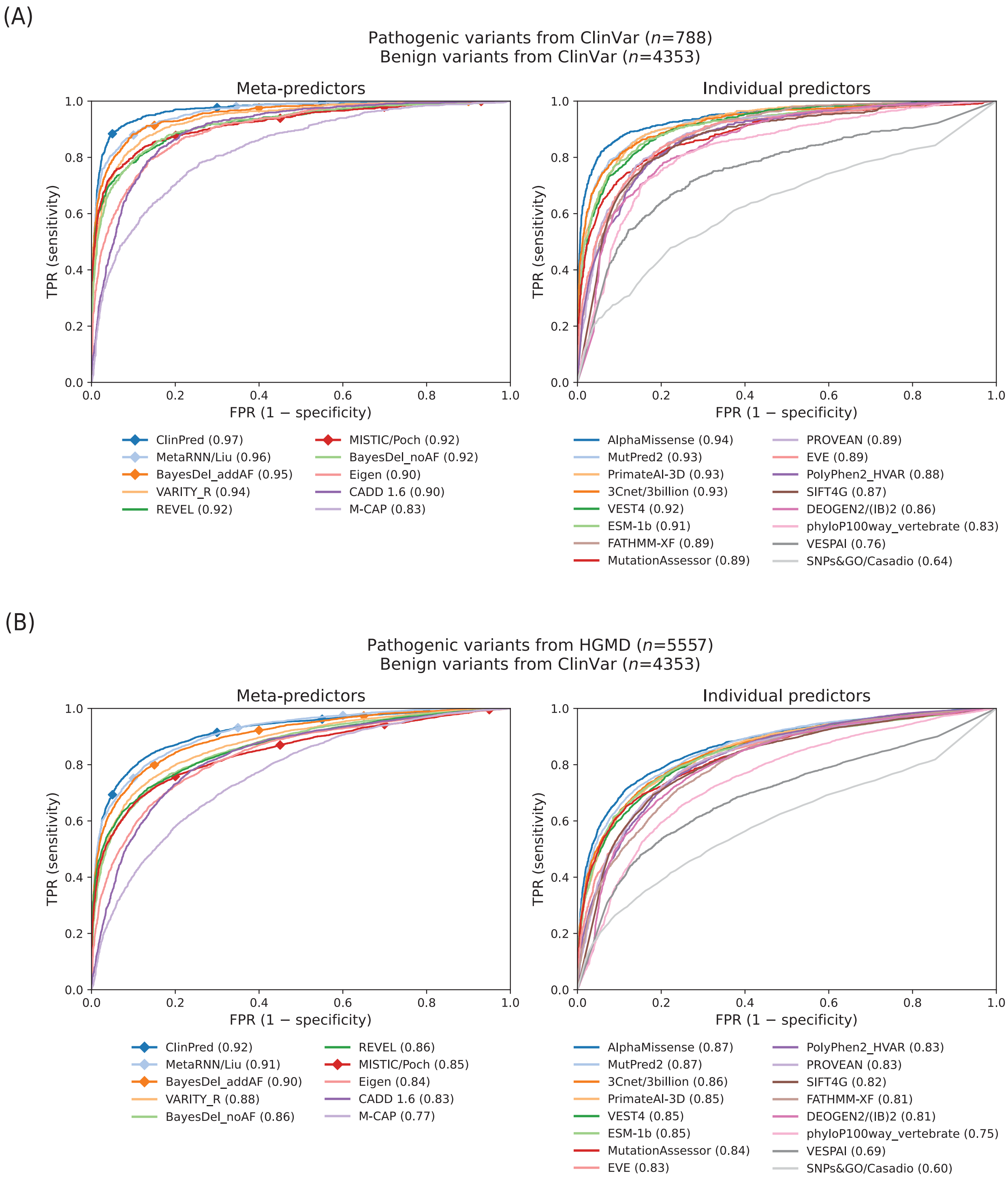

Fig. S5: **Performance when evaluating on only ClinVar or only HGMD pathogenic variants.** We show the ROC curves and AUROCs for meta-predictors (left) and individual predictors (right) using pathogenic variants from either (A) ClinVar or (B) HGMD. Benign variants remain the same (from ClinVar) in both cases. Predictors marked by diamonds use allele frequency as a feature.

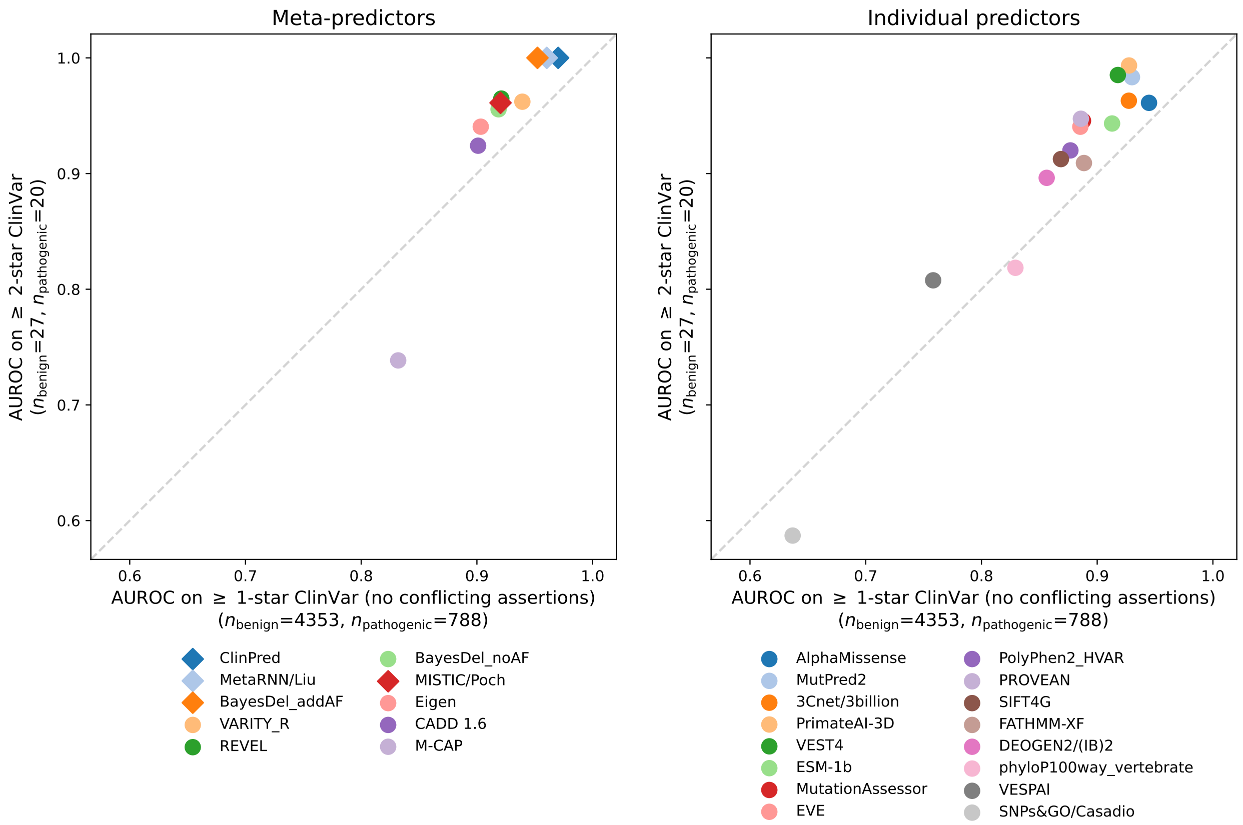

Fig. S6: **Performance on two-star ClinVar variants.** We compare model performance on ClinVar variants with two or more stars (y-axis), indicating multiple submitters and no conflicts, to performance on ClinVar variants in the original dataset (x-axis), which also includes one-star, single-submitter variants, with conflicts removed. Predictors marked by diamonds use allele frequency as a feature.

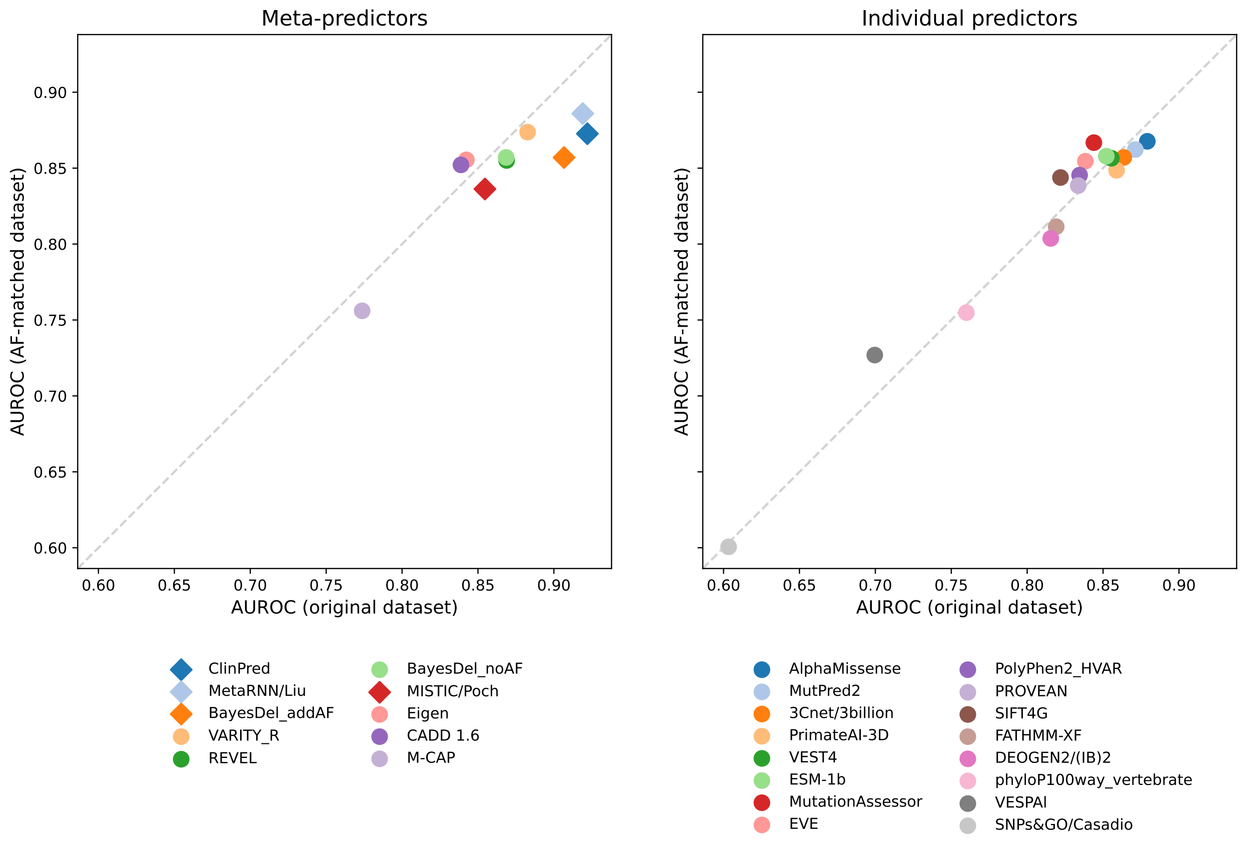

Fig. S7: **Performance on an allele frequency matched dataset.** We created a subset of our evaluation dataset in which the allele frequency (AF) distributions of benign and pathogenic variants are matched. This matching was achieved by equalizing histogram bin counts in the log-transformed allele frequency space, using the Freedman-Diaconis rule [52] to determine bin widths. (See Fig. S1 to visualize the skew in the original dataset). We compare AUROCs on the full dataset (x-axis) to AUROCs on the allele frequency matched dataset (y-axis). Predictors marked by a diamond use allele frequency as a feature.

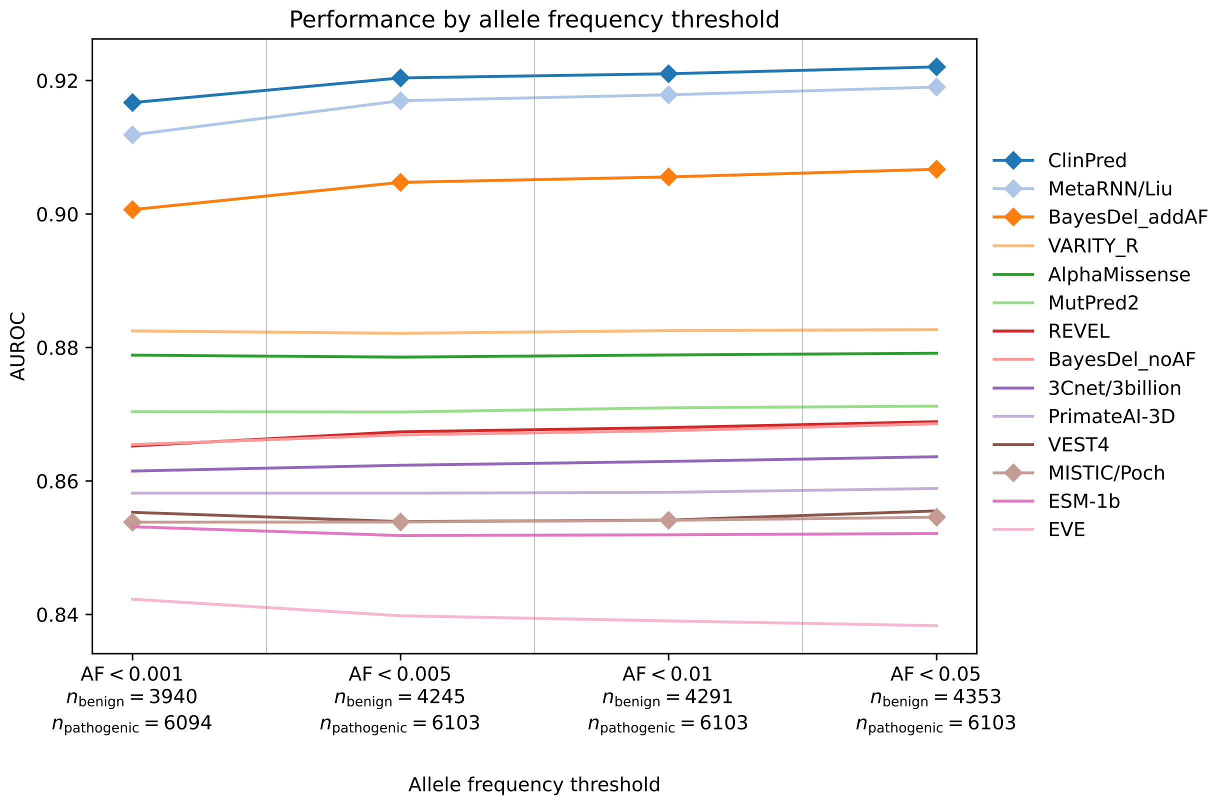

Fig. S8: **Performance by allele frequency threshold.** Model performance is shown as the threshold for defining low-frequency variants used in the evaluation set is adjusted. In contrast to Fig. 3, which uses allele frequency bins, this threshold is an upper bound on the allele frequency and is applied to both benign and pathogenic variants. The rightmost point depicts performance on the original dataset. Predictors marked by a diamond use allele frequency as a feature.

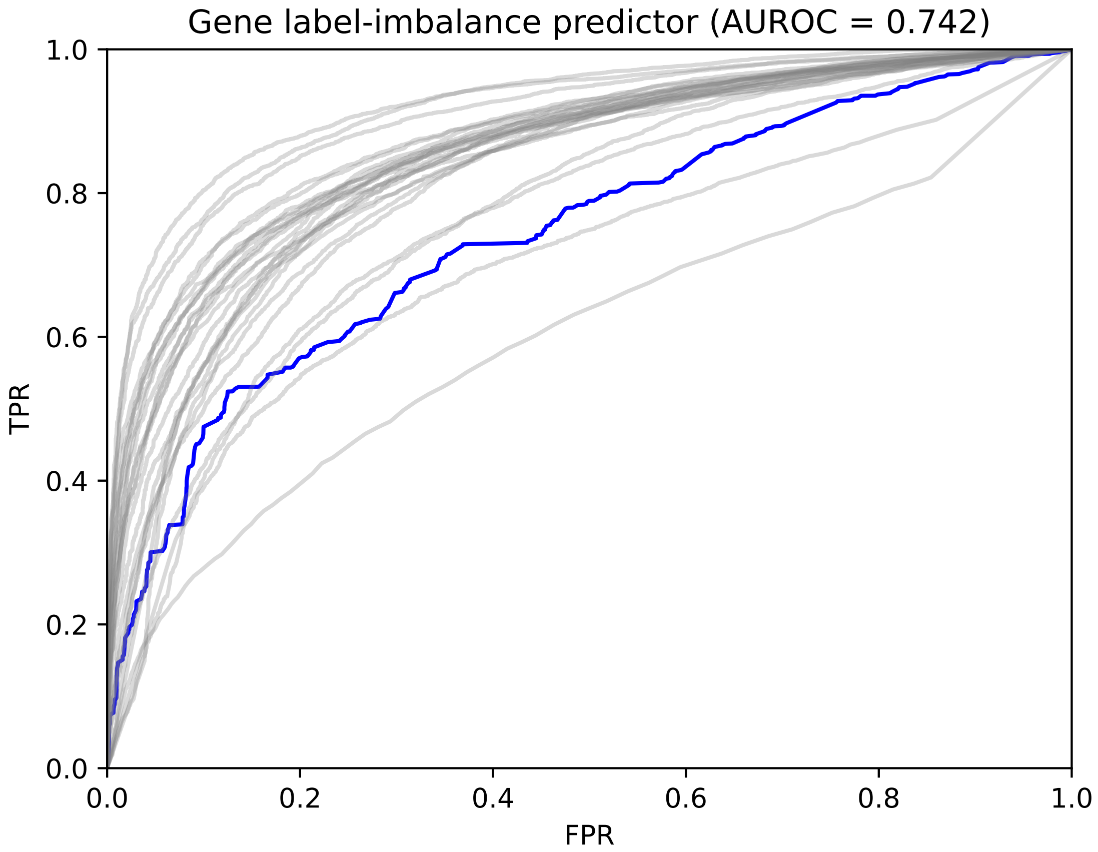

Fig. S9: **Performance of the gene label-imbalance baseline model.** The blue ROC curve for the gene label-imbalance baseline model, which assigns the same score to all variants in a gene (equal to the fraction of ClinVar and HGMD missense variants that were labeled as pathogenic or DM before the cutoff date for our evaluation dataset), is overlayed on top of the light gray ROC curves for all assessed predictors on the full evaluation dataset.

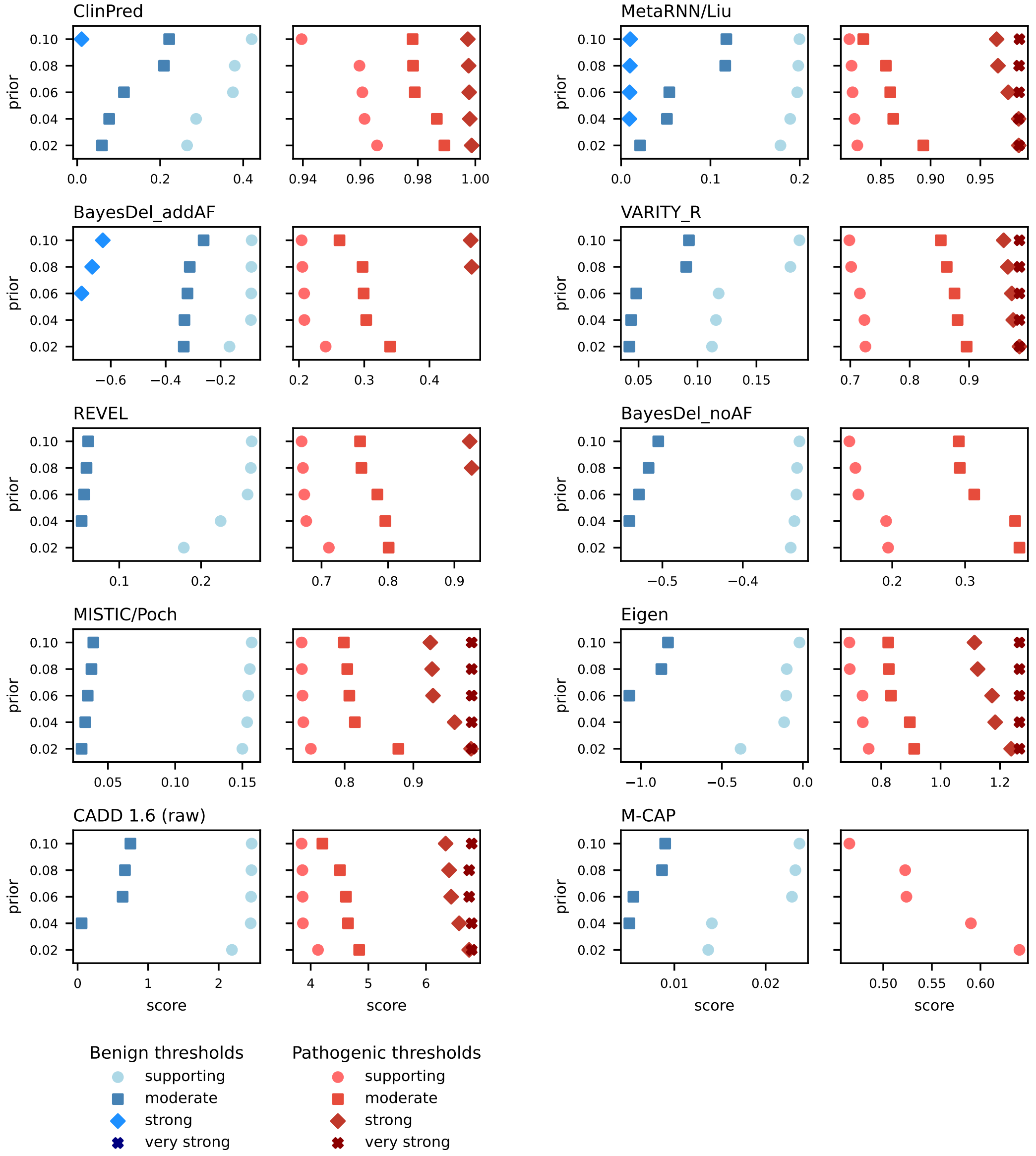

Fig. S10: **Meta-predictor score thresholds for benign and pathogenic evidence strengths at varying prior probabilities of pathogenicity.** We determined the score thresholds that map to PP3 and BP4 evidence strengths by reimplementing the method proposed in [4] in Python. When computing the local posterior probability of pathogenicity (LP) for a given score s, our choice of interval around s differs slightly from that described in [4]. There, the authors recommend choosing an interval that contains the scores of at least 100 dataset variants and at least 3% of low-frequency gnomAD variants. However, since the exact training sets of the predictors assessed here may overlap with gnomAD variants, we remove the gnomAD requirement and increase the number of dataset variants from 100 to 200 for smoothness of LP values.

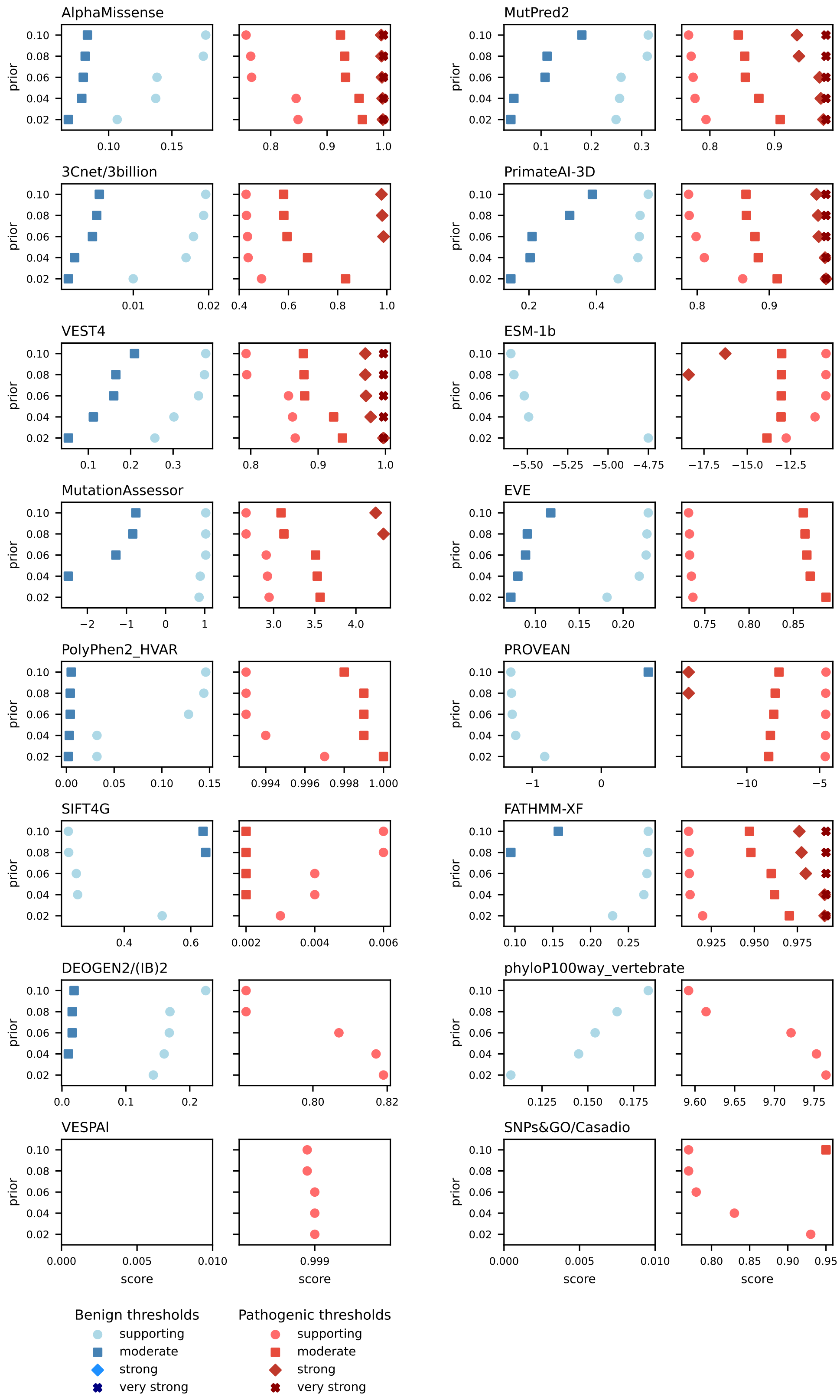

Fig. S11: **Individual predictor score thresholds for benign and pathogenic evidence strengths at varying prior probabilities of pathogenicity.** We determined score thresholds as described in Fig. S10 above.

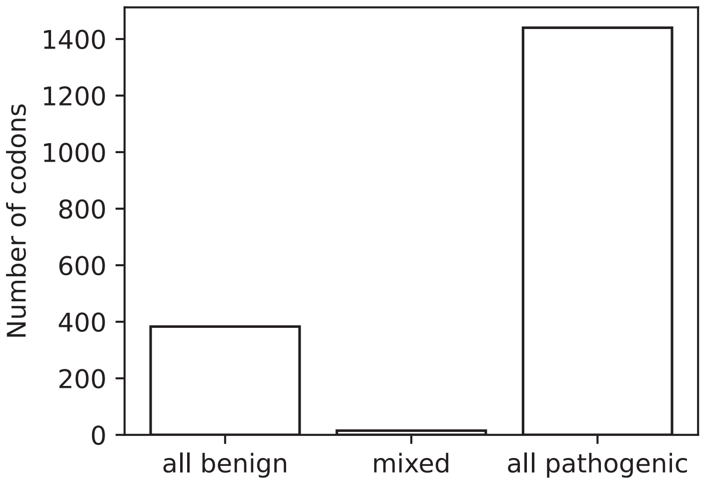

Fig. S12: **Codons with multiple missense variants.** We used SnpEff [48] to determine the affected codon for all high-confidence missense variants in the April 4, 2023 version of ClinVar. We find that variants affecting the same codon are almost always classified in ClinVar as all benign or all pathogenic.

predictor	full-dataset AUROC±SD (rank)	high-specificity AUROC±SD (rank)	high-sensitivity AUROC±SD (rank)	average rank
ClinPred [25]	0.922 ± 0.003 (1)	0.775 ± 0.008 (1)	0.663 ± 0.009 (2)	1.3
MetaRNN/Liu [17]	0.919 ± 0.002 (2)	0.771 ± 0.007 (2)	0.697 ± 0.007 (1)	1.7
BayesDel_addAF [23]	0.907 ± 0.003 (3)	0.757 ± 0.007 (3)	0.653 ± 0.007 (3)	3.0
VARITY_R [40]	0.883 ± 0.003 (4)	0.716 ± 0.007 (7)	0.621 ± 0.007 (5)	5.3
VARITY_R_LOO [40]	0.883 ± 0.003 (5)	0.716 ± 0.008 (6)	0.621 ± 0.007 (6)	5.7
AlphaMissense [36]	0.879 ± 0.003 (6)	0.716 ± 0.007 (8)	0.611 ± 0.006 (7)	7.0
VARITY_ER [40]	0.875 ± 0.003 (7)	0.713 ± 0.006 (10)	0.610 ± 0.007 (8)	8.3
VARITY_ER_LOO [40]	0.875 ± 0.003 (8)	0.713 ± 0.007 (9)	0.609 ± 0.007 (9)	8.7
MutPred2 [29]	0.871 ± 0.003 (9)	0.697 ± 0.007 (12)	0.608 ± 0.007 (11)	10.7
REVEL [33]	0.869 ± 0.003 (10)	0.732 ± 0.006 (4)	0.593 ± 0.006 (21)	11.7
BayesDel_noAF [23]	0.869 ± 0.003 (11)	0.702 ± 0.007 (11)	0.602 ± 0.006 (13)	11.7
MutPred [53]	0.860 ± 0.004 (14)	0.667 ± 0.009 (19)	0.622 ± 0.007 (4)	12.3
3Cnet/3billion_model1 [16]	0.864 ± 0.003 (12)	0.689 ± 0.007 (15)	0.600 ± 0.006 (14)	13.7
VEST4 [35]	0.856 ± 0.004 (17)	0.673 ± 0.007 (17)	0.602 ± 0.006 (12)	15.3
3Cnet/3billion_model2 [16]	0.855 ± 0.004 (18)	0.691 ± 0.007 (13)	0.596 ± 0.006 (18)	16.3
3Cnet/3billion_model4 [16]	0.863 ± 0.004 (13)	0.690 ± 0.008 (14)	0.588 ± 0.006 (25)	17.3
PrimateAI-3D [39]	0.859 ± 0.004 (16)	0.667 ± 0.007 (20)	0.596 ± 0.006 (17)	17.7
3Cnet/3billion_model5 [16]	0.859 ± 0.004 (15)	0.658 ± 0.007 (24)	0.600 ± 0.007 (15)	18.0
MISTIC/Poch [18]	0.855 ± 0.004 (19)	0.720 ± 0.006 (5)	0.566 ± 0.004 (37)	20.3
3Cnet/3billion_model6 [16]	0.842 ± 0.004 (23)	0.655 ± 0.007 (25)	0.596 ± 0.006 (19)	22.3
3Cnet/3billion_model3 [16]	0.840 ± 0.004 (25)	0.661 ± 0.007 (21)	0.591 ± 0.006 (22)	22.7
PolyPhen2_HVAR [31]	0.835 ± 0.004 (28)	0.612 ± 0.006 (31)	0.609 ± 0.006 (10)	23.0
MVP [54]	0.842 ± 0.004 (24)	0.659 ± 0.007 (22)	0.589 ± 0.006 (24)	23.3
MutationAssessor [28]	0.844 ± 0.004 (21)	0.674 ± 0.007 (16)	0.568 ± 0.006 (35)	24.0
ESM-1b [37]	0.852 ± 0.004 (20)	0.668 ± 0.007 (18)	0.565 ± 0.005 (38)	25.3
Eigen [26]	0.842 ± 0.004 (22)	0.652 ± 0.007 (26)	0.579 ± 0.006 (28)	25.3
EVE [38]	0.838 ± 0.005 (27)	0.633 ± 0.009 (28)	0.589 ± 0.007 (23)	26.0
DEOGEN2 [21]	0.832 ± 0.004 (30)	0.658 ± 0.006 (23)	0.577 ± 0.005 (32)	28.3
FATHMM-XF [27]	0.819 ± 0.004 (33)	0.607 ± 0.006 (32)	0.595 ± 0.006 (20)	28.3
CADD 1.6 [24]	0.839 ± 0.004 (26)	0.597 ± 0.006 (34)	0.587 ± 0.005 (26)	28.7
PROVEAN [22]	0.834 ± 0.004 (29)	0.603 ± 0.007 (33)	0.578 ± 0.006 (29)	30.3
PolyPhen2_HDIV [31]	0.812 ± 0.004 (36)	0.558 ± 0.004 (41)	0.598 ± 0.006 (16)	31.0
Eigen-PC [26]	0.821 ± 0.004 (32)	0.622 ± 0.007 (30)	0.568 ± 0.005 (36)	32.7
MetaLR [55]	0.798 ± 0.004 (39)	0.629 ± 0.005 (29)	0.578 ± 0.005 (31)	33.0
SIFT4G [34]	0.822 ± 0.004 (31)	0.572 ± 0.007 (38)	0.570 ± 0.005 (34)	34.3
DEOGEN2/(IB)2 [21]	0.816 ± 0.004 (34)	0.544 ± 0.007 (46)	0.584 ± 0.006 (27)	35.7
M-CAP [30]	0.774 ± 0.005 (40)	0.573 ± 0.005 (37)	0.575 ± 0.004 (33)	36.7
MetaSVM [55]	0.813 ± 0.004 (35)	0.637 ± 0.006 (27)	0.531 ± 0.004 (53)	38.3
SIFT [56]	0.802 ± 0.004 (38)	0.565 ± 0.005 (40)	0.561 ± 0.005 (40)	39.3
SNPMuSiC/(IB)2 [57]	0.804 ± 0.004 (37)	0.567 ± 0.004 (39)	0.551 ± 0.003 (44)	40.0
LIST-S2 [58]	0.756 ± 0.005 (43)	0.580 ± 0.005 (35)	0.548 ± 0.004 (47)	41.7
DANN [59]	0.743 ± 0.005 (44)	0.517 ± 0.003 (52)	0.578 ± 0.005 (30)	42.0
MutationTaster [60]	0.757 ± 0.005 (42)	0.522 ± 0.001 (48)	0.562 ± 0.005 (39)	43.0
PrimateAI [50]	0.734 ± 0.005 (46)	0.549 ± 0.005 (43)	0.550 ± 0.004 (45)	44.7
phyloP100way_vertebrate [32]	0.760 ± 0.005 (41)	0.539 ± 0.004 (47)	0.543 ± 0.003 (48)	45.3
LRT [61]	0.737 ± 0.005 (45)	0.521 ± 0.001 (49)	0.553 ± 0.005 (42)	45.3
FATHMM-MKL [62]	0.733 ± 0.005 (47)	0.521 ± 0.003 (50)	0.559 ± 0.004 (41)	46.0
MPC [63]	0.712 ± 0.005 (48)	0.577 ± 0.005 (36)	0.525 ± 0.004 (55)	46.3
FATHMM [64]	0.677 ± 0.005 (51)	0.551 ± 0.004 (42)	0.532 ± 0.003 (51)	48.0
phastCons100way_vertebrate [65]	0.674 ± 0.005 (52)	0.508 ± 0.000 (55)	0.552 ± 0.004 (43)	50.0
VESPAl [20]	0.700 ± 0.005 (49)	0.545 ± 0.004 (45)	0.506 ± 0.001 (58)	50.7
SiPhy_29way_logOdds [66]	0.683 ± 0.005 (50)	0.507 ± 0.002 (56)	0.549 ± 0.004 (46)	50.7
GERP++_RS [67]	0.668 ± 0.006 (53)	0.509 ± 0.002 (54)	0.538 ± 0.004 (49)	52.0
phyloP30way_mammalian [32]	0.627 ± 0.005 (55)	0.509 ± 0.001 (53)	0.536 ± 0.003 (50)	52.7
SNPs&GO/Casadio [19]	0.603 ± 0.005 (56)	0.548 ± 0.004 (44)	0.498 ± 0.000 (60)	53.3
GenoCanyon [68]	0.645 ± 0.005 (54)	0.520 ± 0.002 (51)	0.515 ± 0.002 (57)	54.0
phyloP17way_primate [32]	0.589 ± 0.006 (58)	0.506 ± 0.001 (57)	0.531 ± 0.003 (52)	55.7
phastCons30way_mammalian [65]	0.599 ± 0.006 (57)	0.506 ± 0.001 (58)	0.531 ± 0.003 (54)	56.3
phastCons17way_primate [65]	0.574 ± 0.006 (59)	0.503 ± 0.001 (59)	0.521 ± 0.002 (56)	58.0
bStatistic [69]	0.497 ± 0.006 (60)	0.499 ± 0.001 (60)	0.499 ± 0.001 (59)	59.7

Table S1: **Metrics for all predictors.** For each of the 60 predictors evaluated, we show the AUROC from the full-dataset ROC curve (Fig. 1), as well as the high-specificity and high-sensitivity AUROCs (Fig. 2). We also include the standard deviation of each AUROC (calculated using 1000 bootstrap samples of the evaluation dataset) and the corresponding rank in each category. Predictors are ordered by their average rank across the three metrics. Note that this table combines all evaluated predictors, including meta-predictors and individual predictors, and predictors with and without allele frequency as a feature.
